# Supplementary material for: A profile-based method for identifying functional divergence of orthologous genes in bacterial genomes
Source: Bioinformatics. 2016 Aug 8;32(23):3566–74. doi: 10.1093/bioinformatics/btw518 (PMC5181535; doi:10.1093/bioinformatics/btw518)
Supplement: Supplementary Data [file supp_32_23_3566__index.html]

A profile-based method for identifying functional divergence of orthologous genes in bacterial genomes — A profile-based method for identifying functional divergence of orthologous genes in bacterial genomes — Supplementary Data 

# A profile-based method for identifying functional divergence of orthologous genes in bacterial genomes

## Supplementary Data

files

- Supplementary Data - pdf file
- Supplementary Data - pdf file
