# Supplementary material for: A profile-based method for identifying functional divergence of orthologous genes in bacterial genomes
Source: Bioinformatics. 2016 Aug 8;32(23):3566–74. doi: 10.1093/bioinformatics/btw518 (PMC5181535; doi:10.1093/bioinformatics/btw518)
Supplement: Supplementary Data [file supp_btw518_supplementary-text_final.pdf]

Technical details for: A profile-based method for measuring the impact of genetic variation

Nicole E. Wheeler[1]*, Lars Barquist[2], Robert Kingsley[3], Paul P. Gardner[1,4]

Abstract
In the following we provide some mathematical justification for the Delta bitscore metric that we evaluate in the accompanying manuscript.

Keywords
genome variation — genotype — phenotype

[1] *School of Biological Sciences, University of Canterbury, Christchurch, New Zealand.*
[2] *Institute for Molecular Infection Biology, University of Wuerzburg, Wuerzburg, Germany.*
[3] *Institute of Food Research, Norwich Research Park, Norwich, Norfolk, United Kingdom.*
[4] *Biomolecular Interaction Centre and the Bio-Protection Research Centre, University of Canterbury, Christchurch, New Zealand.*
***Corresponding author**: nicole.wheeler@pg.canterbury.ac.nz

Introduction

In this document we compare the mathematics motivating profile HMM [1, 2] based methods for quantifying the likely phenotypic significance of genetic variation. We focus on methods that compare a reference (ref) sequence to a variant (var) [3, 4, 5, 6, 7, 8] These methods are the *logR.E-value* approach [3], *FATHMM* [4, 5, 6], HMMvar [7, 8] and *DBS* (this study).

1. Methods

Delta bitscore (DBS)

We define delta bitscore (DBS) as:

$$DBS = (x_{ref} - x_{var}) \quad (1)$$

The bitscore (x) for an HMM is defined as a log of probability ratios [2]:

$$x = \log_2 \left(\frac{P(seq|M)}{P(seq|N)} \right) \quad (2)$$

Where M is a profile model derived from a sequence alignment. M can generate and score sequences based upon how likely they are to have been produced by the same process as those in the sequence alignment. N is a null model, that generates and scores sequences based upon how likely they are to have produced by a random process.

Therefore, DBS can be re-written as:

$$\begin{aligned} DBS(seq_{ref}, seq_{var}) &= \log_2 \left(\frac{P(seq_{ref}|M)}{P(seq_{ref}|N)} \right) \\ &- \log_2 \left(\frac{P(seq_{var}|M)}{P(seq_{var}|N)} \right) \quad (3) \\ &\approx \log_2 \left(\frac{P(seq_{ref}|M)}{P(seq_{var}|M)} \right) \quad (4) \end{aligned}$$

If we make the simplifying assumption that the null models for $P(seq_{ref}|N)$ and $P(seq_{var}|N)$ are approximately equal (i.e. equal length and amino acid composition). Therefore, the first term of Equation 8 and Equation 4 are, in most situations, equivalent.

logR.E-value

Clifford *et al.* (2004) suggest using the following measure to estimate the significance of a genetic variant:

$$\log R.E = \log_{10} \left(\frac{E - value_{var}}{E - value_{ref}} \right) \quad (5)$$

Where $E - value_{var}$ and $E - value_{ref}$ correspond to the expectation value derived from HMMER matches (to the same model) for a variant (var) and canonical (can) protein sequence.

$E - values$ are generally estimated by fitting an exponential distribution to an empirical (usually simulated) distribution. I.e.

$$E - value = \kappa M N e^{\lambda x} \quad (6)$$

Where x is the bit-score for a match between a profile HMM and a sequence, MN is the product of the database size

and the model length and, finally κ and λ are parameters that ensure the intercept with the y-axis is correct and that the curve matches an empirical distribution .

In a breakthrough theoretical paper by Sean Eddy [9], he showed that the most computationally expensive parameter to estimate (λ) is a constant i.e. $\lambda = \ln(2)$.

Thus Equation 5 can be rewritten as:

$$\begin{aligned} \log R.E &= \log_{10}\left(e^{\lambda x_{var}}\right) - \log_{10}\left(e^{\lambda x_{ref}}\right) \qquad (7) \\ &= \left(x_{var} - x_{ref}\right) * \log_{10}(e^{\lambda}) \\ &= -DBS * constant \end{aligned}$$

If the base for the exponential and the logarithms had been equal, then *constant* equals λ. In either case, a constant multiplied by the difference between two bitscores is all that remains.

FATHMM

Shihab *et al* (2013) define the following unweighted measure for estimating the significance of a single non-synonymous SNP (the weighted version is trained to discriminate human disease from polymorphic variation, therefore is not directly comparable to our general approach) [4]. Their metric is a logit or log-odds value, comparing the emission probability of the wild-type variant (P_{ref}) and a mutant variant (P_{var}) when the mutant is a single, non-synonymous point mutation (i.e. not multiple point mutations or indels):

$$\begin{aligned} unweighted &= \ln\left(\frac{P_{var}/(1-P_{var})}{P_{ref}/(1-P_{ref})}\right) \\ &= \ln\left(\frac{P_{var}}{P_{ref}}\right) + \ln\left(\frac{1-P_{ref}}{1-P_{var}}\right) \qquad (8) \\ &\approx -DBS + \ln\left(\frac{1-P_{ref}}{1-P_{var}}\right) \qquad (9) \end{aligned}$$

The value $1-P_{ref}$ and $1-P_{var}$ can be re-written as the following summation:

$$1 - P_{ref} = \sum_{i \in amino-acids, i \neq ref} P_i \qquad (10)$$

$$1 - P_{var} = \sum_{j \in amino-acids, j \neq var} P_j \qquad (11)$$

Equations 10&11 share 18 terms (for each of the 20 amino acids, less the ones corresponding to the wild-type (w) and mutant (m) variants. Therefore, $1-P_{ref} \approx 1-P_{var}$ for most realistic biological results. As a consequence, the second term of Equation 9 is approximately zero (or at least, modest in comparison to the first term when there is a large difference between P_{ref} and P_{var}). Therefore a difference between bitscores is the term that dominates Equation 8.

HMMvar

Liu *et al* (2014&2015) present a similar metric to *FATHMM* for estimating the significance of variation using a profile-HMM based approach, with some important differences in the implementation [7, 8]. The authors calculate the probability of each sequence directly from *HMMER3* bitscores using $P = P_{null} * e^B$, where $P_{null} = exp(l * \log(P_1) + \log(1-P_1))$, the sequence length is l and $P_1 = \frac{350}{351}$.

$$\begin{aligned} S &= \frac{P_{ref}/(1-P_{ref})}{P_{var}/(1-P_{var})} \qquad (12) \\ \log_2(S) &= \log_2\left(\frac{P_{ref}/(1-P_{ref})}{P_{var}/(1-P_{var})}\right) \qquad (13) \\ &= \log_2\left(\frac{P_{ref}}{P_{var}}\right) - \\ &\quad \log_2\left(\frac{1-P_{var}}{1-P_{ref}}\right) \\ &\approx -\log_2\left(\frac{1-P_{var}}{1-P_{ref}}\right) - DBS \qquad (14) \end{aligned}$$

Liu *et al* do discuss the possibility of using *DBS*, which they call *D*, they conclude that “prediction results using *D* were not better than for *S*, and hence are not reported here”.

2. Discussion

As a consequence, the measures used by the log*R.E-value* (Equation 7) and the *FATHMM* (Equation 9) approach are approximations to the more direct estimation of significance, *DBS*. In the case of *FATHMM*, only single point mutations are considered, missing the wealth of variation due to insertions, deletions, multiple SNPs and and other larger-scale variants.

The *HMMvar* approach is similar to DBS, this method employs full length HMMs (including indel states), just like *DBS*.

Consequently, *DBS* is a direct measure of the potential impact of genetic variation, that can be used on small as well as large and complex variants. We propose that this metric can be used to evaluate both population variation as well as variation between species. The mean of the distribution should be approximately zero, while the variance will increase with increasing phylogenetic distance (and different levels of selection).

One factor that may have an undue influence on *DBS* is in the rare cases where the optimal alignment between the profile and the variant and the profile and the canonical sequence differ. For example, *HMMER3* currently only has a local mode (i.e. no “glocal” option). As a result, split matches can occur, and alignment slippage is also possible, particularly for repetitive sequences.

One way to mitigate these possibilities is to use *Forward Scores*, which rather than reporting just the value for an optimal alignment, reports instead the sum of all possible alignments between a query sequence and the profile model.

References

[1] A Krogh, M Brown, I S Mian, K Sjölander, and D Haussler. Hidden markov models in computational biology. applications to protein modeling. *J Mol Biol*, 235(5):1501–31, Feb 1994.

[2] R. Durbin, S. Eddy, A. Krogh, and G. Mitchison. *Biological sequence analysis*. Press, Cambridge U., 1998.

[3] R J Clifford, M N Edmonson, C Nguyen, and K H Buetow. Large-scale analysis of non-synonymous coding region single nucleotide polymorphisms. *Bioinformatics*, 20(7):1006–14, May 2004.

[4] H A Shihab, J Gough, D N Cooper, P D Stenson, G L Barker, K J Edwards, I N Day, and T R Gaunt. Predicting the functional, molecular, and phenotypic consequences of amino acid substitutions using hidden markov models. *Hum Mutat*, 34(1):57–65, Jan 2013.

[5] H A Shihab, J Gough, D N Cooper, I N Day, and T R Gaunt. Predicting the functional consequences of cancer-associated amino acid substitutions. *Bioinformatics*, 29(12):1504–10, Jun 2013.

[6] H A Shihab, J Gough, M Mort, D N Cooper, I N Day, and T R Gaunt. Ranking non-synonymous single nucleotide polymorphisms based on disease concepts. *Hum Genomics*, 8:11, 2014.

[7] M Liu, L T Watson, and L Zhang. Quantitative prediction of the effect of genetic variation using hidden markov models. *BMC Bioinformatics*, 15:5, 2014.

[8] M Liu, L T Watson, and L Zhang. Hmmvar-func: a new method for predicting the functional outcome of genetic variants. *BMC Bioinformatics*, 16:351, 2015.

[9] S R Eddy. A probabilistic model of local sequence alignment that simplifies statistical significance estimation. *PLoS Comput Biol*, 4(5):e1000069, May 2008.
