# Supplementary material for: A profile-based method for identifying functional divergence of orthologous genes in bacterial genomes
Source: Bioinformatics. 2016 Aug 8;32(23):3566–74. doi: 10.1093/bioinformatics/btw518 (PMC5181535; doi:10.1093/bioinformatics/btw518)
Supplement: Supplementary Data [file supp_btw518_SupplementaryMaterial_edit.pdf]

Supplementary material

Supplementary Figure 1: Workflow diagrams for custom model and Pfam model based proteome analyses

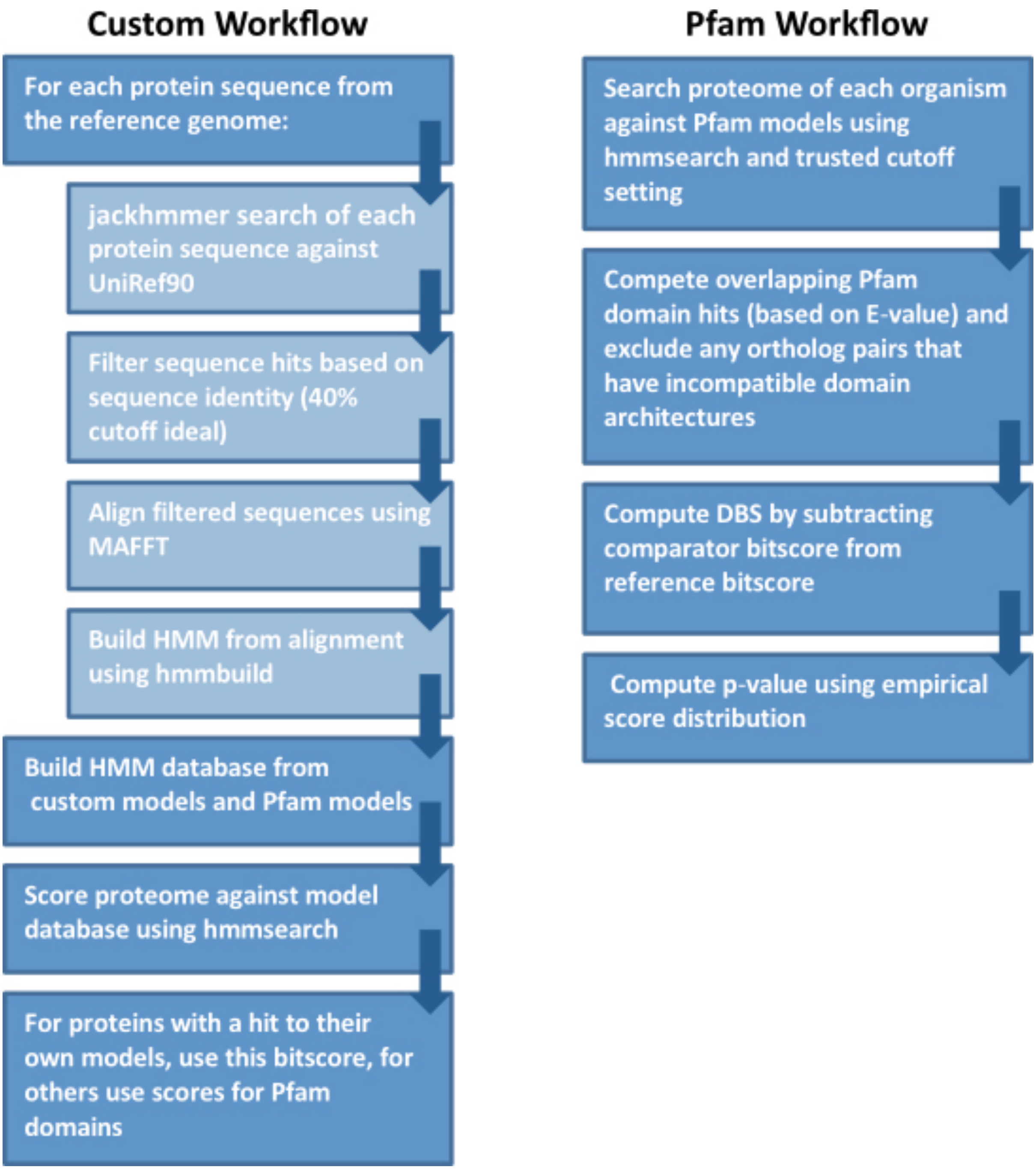

Supplementary Figure 2: AUC values for performance of DBS and other methods using protein mutagenesis data.

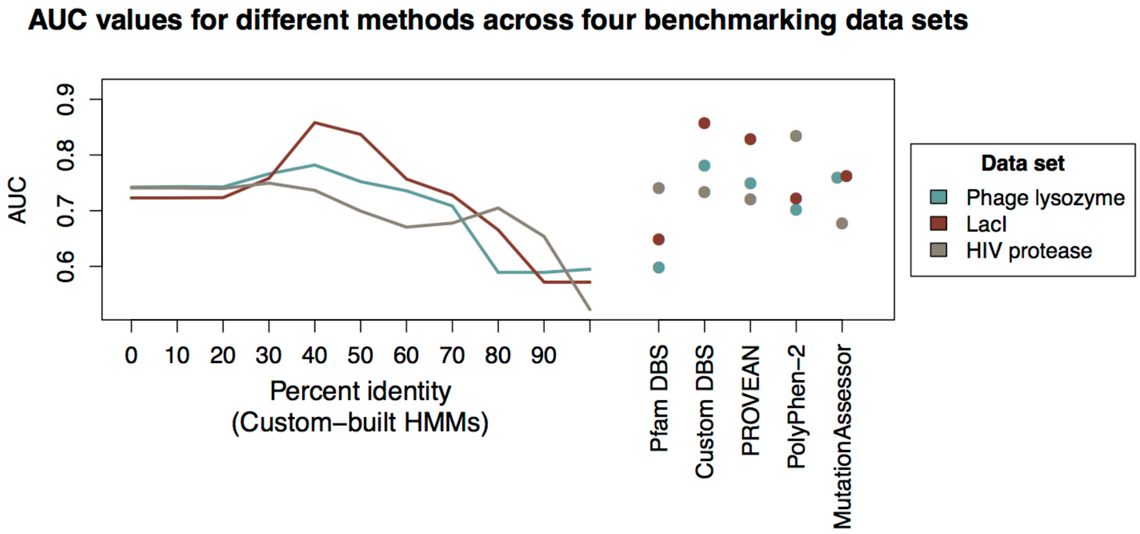

Supplementary Figure 3: ROC curves for performance of DBS and other methods using data from humsavar.

DBS performance is shown for three different sources of HMMs - Pfam domain HMMs, Treefam vertebrate gene HMMs and custom built HMMs (see Proteome Analysis methods for procedure for building custom models). Weighted FATHMM was the best performer, however this has been shown to be largely due to circularity in it's training/scoring scheme, making it a poor predictor of pathogenicity in new variants (Grimm et al. 2015).

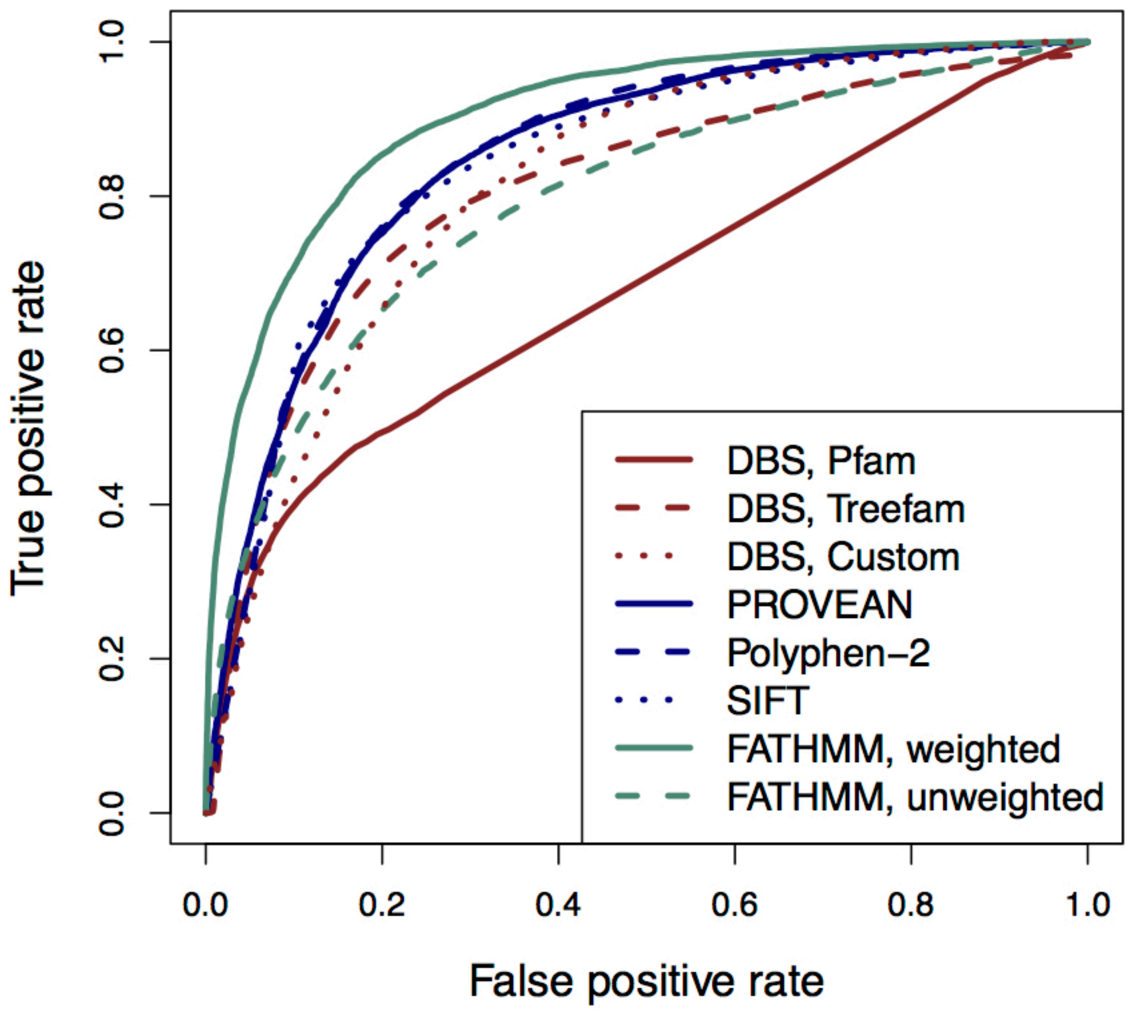

Supplementary Figure 4: Relative cost curves for performance of DBS and other methods using data from humsavar. The curve indicated the relative value of the predictive method over random guessing across a range of costs of a false negative compared to a false positive. Cost for a false positive is set at 1 and the cost of a false negative (c) is shown on a log scale on the x axis.

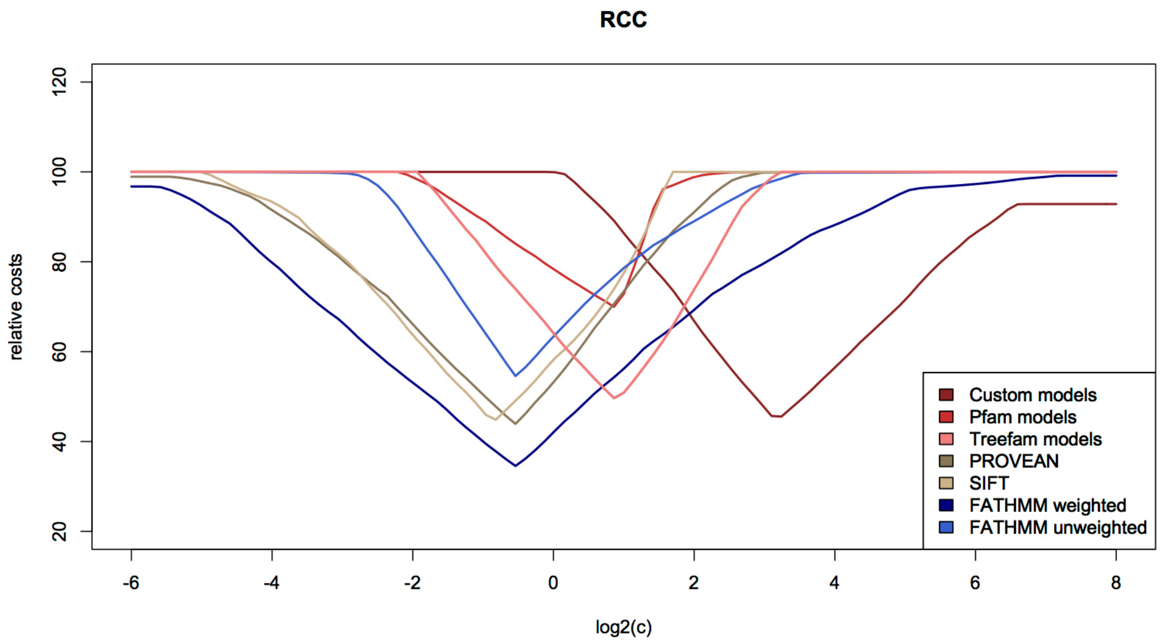

Supplementary Figure 5: Closer view of the ROC curve for the LacI predictions, FPR up to 5%.
Pfam models perform well at low false positive rates, then performance relative to other methods declines with increasingly permissive scoring thresholds.

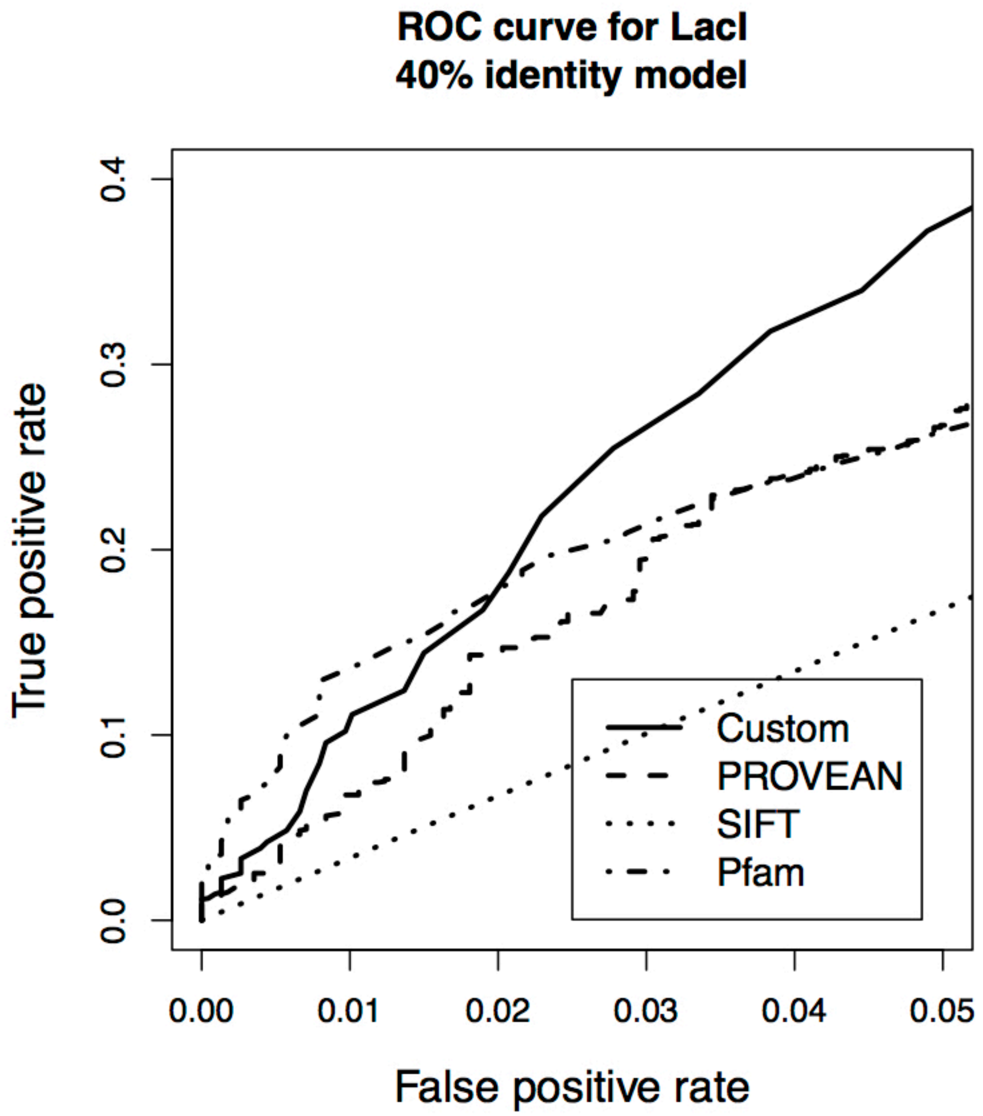

Supplementary Figure 6: Score distributions for selected *Salmonella* pairwise comparisons.

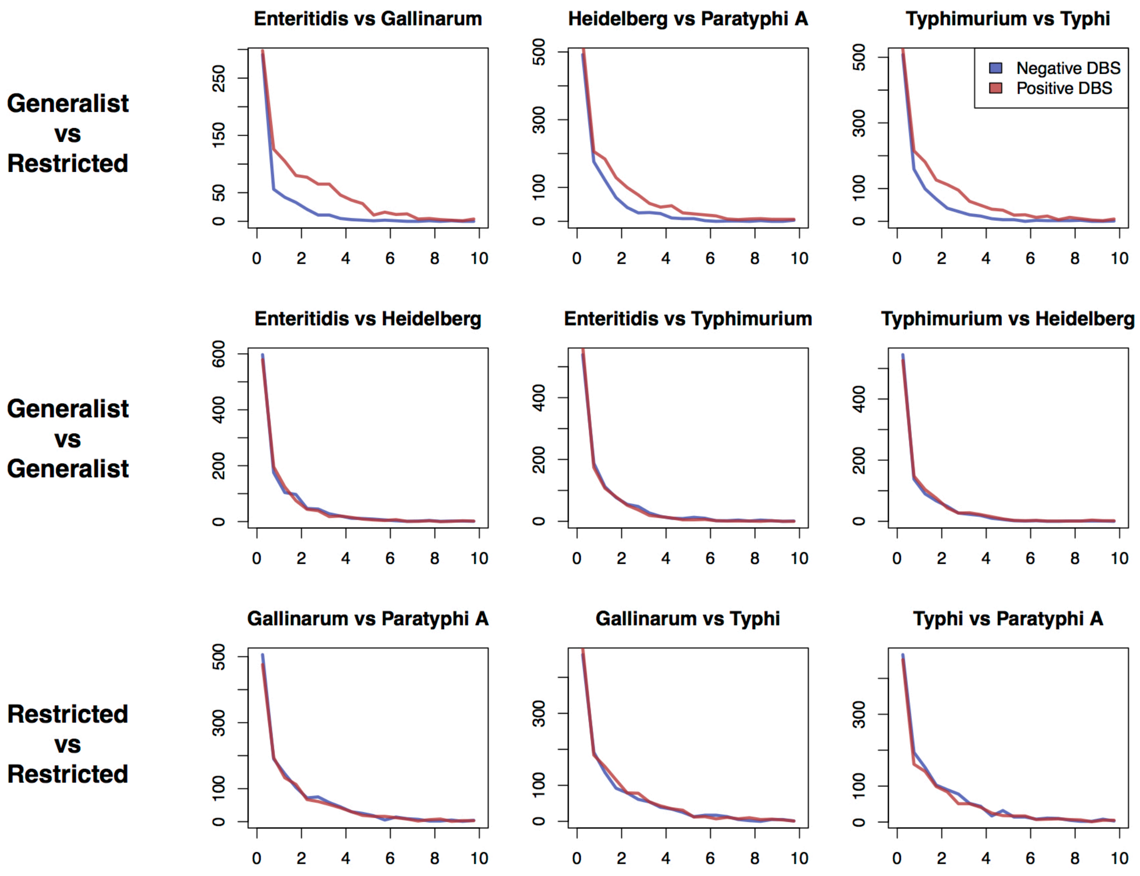

Supplementary Table 1: Performance measures for various predictive methods.

Protein	Method	AAC	Max MCC	Score @ max MCC	Sensitivity @ max MCC	Specificity @ max MCC	PPV @ max MCC	NPV @ max MCC	Max accuracy
LacI	**Custom models**	0.2578	0.600	2.6	0.768	0.831	0.781	0.821	0.804
	**Pfam**	0.0701	0.305	3.4	0.282	0.941	0.790	0.626	0.653
	**PROVEAN**	0.2035	0.535	3.68	0.789	0.750	0.712	0.819	0.770
	**SIFT**	0.0745	0.352	0.01	0.596	0.752	0.653	0.704	0.683
	**Mutation Assessor**	0.1286	0.450	2.08	0.722	0.731	0.678	0.770	0.727
Lysozyme	**Custom models**	0.1437	0.462	3.6	0.558	0.875	0.674	0.810	0.775
	**Pfam**	0.0377	0.227	3.6	0.176	0.956	0.651	0.715	0.712
	**PROVEAN**	0.1141	0.364	4.7	0.707	0.680	0.506	0.833	0.730
	**SIFT**	0.0592	0.299	0.01	0.415	0.834	0.538	0.755	0.709
	**Mutation Assessor**	0.1178	0.411	2.41	0.591	0.813	0.595	0.811	0.743
HIV protease	**Custom models**	0.1498	0.376	1.3	0.969	0.261	0.727	0.806	0.744
	**Pfam**	0.1416	0.382	1.6	0.636	0.748	0.836	0.503	0.723
	**PROVEAN**	0.1635	0.393	3.937	0.880	0.468	0.770	0.658	0.747
	**SIFT**	0.2520	0.531	0.12	0.893	0.595	0.817	0.733	0.801
	**Mutation Assessor**	0.1204	0.259	3.03	0.788	0.459	0.745	0.520	0.691

Supplementary Table 2: AUC values for custom model predictions when proteins are split into groups according to the number of sequences used to build the model, or effective sequence number calculated for the model.

Discriminatory factor	Class	Total count	Polymorphism	Disease	AAC
**Effective Sequence Number**	x<0.5	10454	9609	845	0.128
	0.5≤x<0.75	9117	8307	810	0.112
	0.75≤x<1	1963	1809	154	0.168
	1≤x	1344	1296	48	0.084
**Number of sequences**	x≤3	1938	1895	43	0.076
	3≤x<5	9262	8699	563	0.135
	5≤x<7	3610	3354	256	0.130
	7≤x<10	2937	2592	345	0.117
	10≤x	6574	5915	659	0.119

Relative cost curves are shifted to the right for human data due to the low frequency of disease associated variants, therefore AAC was calculated across the range -6 – 8 for log2(c). AAC across the same range for Pfam models was 0.041.

Supplementary Table 3: HDCs from Nuccio and Bäumler’s analysis that were excluded from our scoring for having no hits to the Pfam database.

All Enteritidis genes listed also had no Pfam hit for the Gallinarum ortholog. All Gallinarum genes had a hit for the Enteritidis ortholog.
HP = hypothetical protein, CHP = conserved hypothetical protein, SP = signalling peptide

Gene ID	Description
SEN0018	Long, known protein
SEN0272	Short HP
SEN0324	Short HP
SEN0325	Short SP
SEN0490	Short SP
SEN0966	Short SP
SEN1000	Short SP
SEN1006	Short HP
SEN1648	Short HP
SEN1469	Short SP
SEN1362	Short SP
SEN1335	Long HP
SEN1331	Short CHP
SEN1066	Short CHP
SEN2203	Short CHP
SEN2997	Short exported protein
SEN3537	Short known protein
SEN3584	Short HP
SEN3981	Long HP
SEN4102	Short HP
SEN4264	Short HP
SG0018	Pseudogene
SG0203	Pseudogene
SG0374	Pseudogene
SG0501	Pseudogene
SG0658	Pseudogene
SG0818	Pseudogene
SG0863	Pseudogene
SG0884	Pseudogene
SG1909	Pseudogene
SG1878	Pseudogene
SG1863	Pseudogene
SG1824	Pseudogene
SG1762	Pseudogene
SG1451	Pseudogene
SG1325	Pseudogene
SG1239	Pseudogene
SG2070	Pseudogene
SG2096	Pseudogene
SG2166	Pseudogene
SG2192	Pseudogene
SG2268	Pseudogene
SG2332	Pseudogene
SG2583	Pseudogene
SG2684	Pseudogene
SG2847	Pseudogene
SG3830	Pseudogene
SG3809	Pseudogene
SG3782	Pseudogene
SG3421	Pseudogene
SG3297	Pseudogene
SG4321	Pseudogene
SG4334	Pseudogene

SG4355	Pseudogene
SG4397	Pseudogene
SG4413	Pseudogene

Supplementary Table 4: HACs from Nuccio and Bäumler's analysis that failed to reach our HAC scoring cutoff.

**Gene name (*S.* Enteritidis)**	**Description**
SEN0240	Conserved alternate start
SEN0340	Premature stop
SEN0373	Alternate start
SEN0495	Premature stop
SEN0618	Start site varies across Salmonella strains
SEN0994	Premature stop
SEN1911	Premature stop
SEN1884	Alternate start
SEN1865	Premature stop
SEN1756	Premature stop
SEN1698	Conserved alternate start
SEN1661	Premature stop
SEN1375	Alternate start
SEN1363	Premature stop
SEN1279	Premature stop
SEN1234	Premature stop
SEN1979	Premature stop
SEN1981	Premature stop
SEN2034	Premature stop
SEN2553	Premature stop
SEN2611	Premature stop
SEN3115	Indel
SEN3125	Premature stop
SEN3636	Alternate start
SEN3737	Indel
SEN3830	Indel
SEN4011	2AA premature stop
SEN4271	Alternate start

Supplementary Table 5: Putative HACs in genes involved in anaerobic metabolism of *S.* Enteritidis and *S.* Gallinarum (according to the classification in Table S7 of Nuccio and Bäumler, 2014, using our scoring method).

No Enteritidis anaerobic metabolism genes reached the significance level for LOF. Pfam models are only listed if they gave a non-zero DBS.

Locus tag (E)	Locus tag (G)	Model	DBS	Gene name	Gene product	Nature of mutation
SEN1744	SG1817	SEN1744	636.5	gdhA	NADP-specific glutamate dehydrogenase	Truncation
SEN3067	SG3121	SEN3067	547.6	ygjU	probable membrane transport protein	Truncation
SEN2182	SG2225	SEN2182	516	mglA	galactoside transport atp-binding protein mgla	Truncation
SEN3696	SG3551	SEN3696	515.9	rbsA	high affinity ribose transport protein	Truncation
SEN3081	SG3136	SEN3081	495.9	tdcG	L-serine dehydratase	Truncation
SEN2805	SG2870	SEN2805	437.5	gudD	glucarate dehydratase protein	Truncation
SEN1739	SG1812	SEN1739	383.8	astA	arginine N-succinyltransferase	Truncation
SEN0775	SG0808	SEN0775	314.7	glnH	glutamine-binding periplasmic protein precursor	Truncation
SEN4072	SG4145	SEN4072	312.2	fumB	fumarate hydratase class I	Truncation
SEN2018	SG2044	SEN2018	293.1	cbiO	putative cobalt transport ATP-binding protein	Truncation
SEN2860	SG2926	SEN2860	292.6	kduD	2-keto-3-deoxygluconate oxidoreductase	Truncation
SEN1084	SG1132	SEN1084	290.6	cheM	methyl-accepting chemotaxis protein II	Truncation
SEN4073	SG4146	DcuA_DcuB	244.5	dcuB	anaerobic C4-dicarboxylate transporter	Truncation
SEN1543A	SG1612	SEN1543A	168		putative membrane transport protein	Truncation
SEN3840	SG3372	SEN3840	142.6	rhaR	L-rhamnose operon transcriptional activator	Truncation
SEN2030	SG2057	CbiD	127.1	cbiD	cobalt-precorrin-6A synthase [deacetylating]	Truncation
SEN3514	SG3738A	SEN3514	109.2	lldP	L-lactate permease	Frameshift
SEN2031	SG2058	SEN2031	91.7	cbiC	precorrin-8X methylmutase	Truncation
SEN2048	SG2079	PF01923.13	78.5	pduO	propanediol utilization protein	Truncation
SEN2371	SG2419	SEN2371	49.7	yfcY	putative 3-ketoacyl-CoA thiolase	Alternate start
SEN3496	SG3756	FGGY_N	45.7	lyxK	putative L-xylulose kinase	Truncation
SEN2024	SG2051	CbiJ	34.1	cbiJ	cobalt-precorrin-6a reductase (ec 1.3.1.-)	Alternate start, point mutation
SEN0613	SG0648	Aminotran_1_2	20.4	cobD	aminotransferase	Truncation
SEN2636	SG2698	SEN2636	18.1	gabT	4-aminobutyrate aminotransferase	Indels, point mutations
SEN2038	SG2067	Dehydratase_LU	16	pduC	glycerol dehydratase large subunit	Indels, point mutations
SEN4204	SG4279	SEN4204	14.4	treC	trehalose-6-phosphate hydrolase	Truncation
SEN1477	SG1549	SEN1477	12	narY	respiratory nitrate reductase 2 beta chain	Point mutations
SEN3490	SG3763	Ldh_2	10.9	yiaK	putative carboxylic acid dehydrogenase	Indel
SEN2017	SG2043	SEN2017	10.5	cbiP	putative cobyric acid synthase	Indels, point mutations
SEN0634	SG0669	SBP_bac_3	10	gltI	ABC transporter periplasmic binding protein(glutamate/aspartate?)	Truncation
SEN0826	SG0861	SEN0826	8.8	potI	putrescine transport system permease protein PotI	Indels, point mutations
SEN3603	SG3647	SEN3603	8.2	uhpC	regulatory protein	Point mutations
SEN2230	SG2275	CcmH	7.1	ccmH	cytochrome c-type biogenesis protein H1	Indel
SEN3628	SG3621	CcmH	7.1		cytochrome C-type biogenesis protein	Indel
SEN0506	SG0537	SEN0506	6.9	glxK	glycerate kinase	Point mutations
SEN2334	SG2381	SEN2334	6.3	hisM	histidine transport system permease	Point mutations
SEN2229	SG2274	SEN2229	6.2	narP	nitrate/nitrite response regulator protein NarP	Alternate start, point mutations
SEN1315	SG1396	SEN1315	6.2	btuR	COB(I) alamin adenosyltransferase	Indel
SEN2023	SG2050	CbiK	6.1	cbiK	sirohydrochlorin cobaltochelatase (ec 4.99.1.3)	Point mutation

SEN1276	SG1354	SEN1276	5.6	narH	respiratory nitrate reductase 1 beta chain	Point mutations
SEN3643	SG3605	SEN3643	5.3	dgoT	d-galactonate transporter	Alternate start, point mutations
SEN2337	SG2384	SEN2337	5.2	argT	lysine-arginine-ornithine-binding periplasmic protein precursor	Point mutations
SEN0656	SG0689	LysR_substrate, HTH_1	4.9		lysR-family transcriptional regulator	Point mutation
SEN3088	SG3144	SEN3088	4.9	garR	tartronate semialdehyde reductase (tsar)	Point mutation
SEN1458	SG1526	SEN1458	4.8	ydcW	putative aldehyde dehydrogenase	Point mutation
SEN0105	SG0105	AraC_binding	4.7	araC	arabinose operon regulatory protein	Point mutation
SEN0653	SG0686	SEN0653	4.5	citA	citrate-proton symporter	Point mutations
SEN3826	SG3387	SEN3826	4.5	fdoG	formate dehydrogenase-O, major subunit	Point mutations
SEN3092	SG3148	Tagatose_6_P_K	4.5		putative sugar kinase	Point mutation
SEN3908	SG3303	PFL	4.5	pflD	formate acetyltransferase 2 (ec 2.3.1.54)(pyruvate formate-lyase 2)	Point mutation
SEN2438	SG2489	SEN2438	4.3	eutB	ethanolamine ammonia-lyase heavy chain	Point mutation
SEN2014	SG2040	SEN2014	4.2	cobT	Nicotinate-nucleotide--dimethylbenzimidazolephosphoribosyltransferase	Point mutations
SEN2036	SG2065	SEN2036	3.9	pduA	putative propanediol utilization protein	Point mutation
SEN4147	SG4223	DeoRC	3.9	yjfQ	probable transcriptional regulator	Point mutation
SEN2055	SG2086	SEN2055	3.9	pduW	Acetokinase.	Point mutation
